# The Power of Old Hats: Rediscovering Inosine-EpPCR to Create Starting Libraries for Whole-Cell-SELEX

**DOI:** 10.3390/bios15070448

**Published:** 2025-07-12

**Authors:** Grigory Bolotnikov, Ann-Kathrin Kissmann, Daniel Gruber, Andreas Bellmann, Roger Hasler, Christoph Kleber, Wolfgang Knoll, Frank Rosenau

**Affiliations:** 1 Institute of Pharmaceutical Biotechnology, Ulm University, 89081 Ulm, Germany; grigory.bolotnikov@uni-ulm.de (G.B.); ann-kathrin.kissmann@uni-ulm.de (A.-K.K.); daniel.gruber@uni-ulm.de (D.G.); andreas.bellmann@uni-ulm.de (A.B.); 2 Faculty of Medicine and Dentistry, Danube Private University, Steiner Landstraße 124, 3500 Krems an der Donau, Austria; roger.hasler@dp-uni.ac.at (R.H.); christoph.kleber@dp-uni.ac.at (C.K.); wolfgang.knoll@dp-uni.ac.at (W.K.)

**Keywords:** DNA aptamer, Cell-SELEX, biosensors, polyclonal library

## Abstract

Shaking off the forgetfulness towards the methodological power of inosine-mediated error-prone PCR (epPCR), this study reintroduces an often-underappreciated method as a considerably powerful approach for generating aptamer libraries from a single decameric ATCG-repeat-oligonucleotide. The aim was to demonstrate that this simple way of creating sequence diversity was suitable for delivering functional starting libraries for a set of ten whole-cell-SELEX (Systematic Evolution of Ligands by Exponential Enrichment) processes. This epPCR method uses inosine to introduce targeted mutations, avoiding the need for commercial oligo pools or large-scale synthesis. We applied this method to a “universal aptamer” and subjected the three resulting libraries to two rounds of selection against ten diverse targets including probiotic and pathogenic bacteria (Gram-negative and -positive) as well as human cell lines. The enriched aptamers exhibited new binding specificities, demonstrating that the approach supports functional selection. Much like dusting off an old tool and finding it perfectly suited for a modern task, this work shows that revisiting established techniques can address current challenges in aptamer development. Our main finding is that epPCR provides a robust, cost-effective strategy for generating starting libraries and lowers the barrier for initiating successful SELEX campaigns.

## 1. Introduction

The invention of the polymerase chain reaction (PCR) in 1983 by Kary Mullis sparked the “Polymerase Chain Revolution”, fundamentally transforming molecular biology by enabling rapid and efficient DNA amplification [1]. PCR has since become a “workhorse” method of modern science, widely used in medical diagnostics to detect infections and genetic disorders, as well as in genetic engineering for gene editing and the creation of genetically modified organisms [2,3,4,5,6,7,8]. It also plays a pivotal role in forensic science [9], agriculture [10,11], and environmental monitoring [12,13,14], enabling precise genetic analysis. The PCR market is projected to be valued at 14.43 billion USD in 2025 [15], underscoring its widespread application and decades of enduring impact. While conventional PCR is optimized for fidelity [16], error-prone PCR (epPCR) is deliberately engineered to create “controlled chaos” on the product’s sequence level, exploiting polymerase infidelity to generate diversity. The aim is to generate a heterogeneous pool of DNA variants from a single template, which can be screened for desirable traits in downstream applications such as directed evolution, functional genomics, and aptamer development [17].

Early developments in epPCR emerged in 1992 with the use of low-fidelity DNA polymerases, as demonstrated by Cadwell and Joyce, who pioneered its application for mutagenesis [18]. Around the same time, Oskar Kuipers introduced the incorporation of deoxyinosine triphosphate (dITP) in 1993 to further modulate mutation rates [19].

Frances H. Arnold played a pivotal role in utilizing epPCR as a central tool for directed evolution in 1994, a methodology that ultimately earned her the Nobel Prize in Chemistry [20]. Parallel advancements by Willem P.C. Stemmer in 1994 led to DNA shuffling, an approach that combined epPCR-induced mutations with the recombination of homologous genes to enhance genetic diversity [21].

In 1994, Jack W. Szostak further refined directed evolution techniques, incorporating epPCR as a means of selection of catalytic RNAs [22]. Later developments expanded epPCR applications, such as David Ostermeier’s integration of phage display for evolving enzyme variants in 1999 [23], and M. T. Reetz and K.E. Jaeger’s application of epPCR in 2000 to systematically mutate active-site residues, leading to enzyme optimization [24].

**Figure 1:**
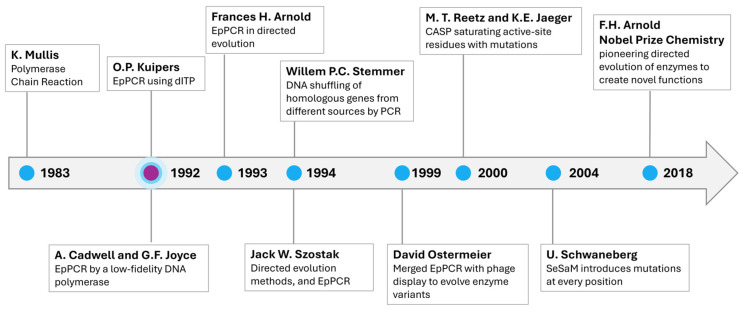
Timeline of key developments in error-prone PCR (epPCR) and directed evolution. Major milestones are shown from the invention of PCR in 1983 by Kary Mullis to the 2018 Nobel Prize in Chemistry awarded to Frances H. Arnold for her work in directed enzyme evolution.

Further advancements included Schwaneberg’s introduction of Sequence Saturation Mutagenesis (SeSaM) in 2004, a method enabling mutations at every position within a sequence, expanding the potential of epPCR for protein engineering [25]. Collectively, these innovations have cemented epPCR as a fundamental tool in molecular evolution over the last four decades (Figure 1).

Aptamers, introduced in 1990 by Ellington and Szostak [26], as well as Tuerk and Gold [27], are short, single-stranded oligonucleotides that present a promising alternative to antibodies and their derivatives [28]. Unlike antibodies, aptamers offer several advantages, including remarkable chemical and physical stability [29,30,31], the ability to be regenerated without losing functionality, and ease of modification [32]. They can be synthesized in large quantities in vitro and exhibit low immunogenicity, making them highly attractive for various applications [33]. Furthermore, aptamers undergo molecular evolution entirely in vitro, starting from synthetic random oligonucleotide libraries with sequence spaces typically including up to 10^16^ distinct sequences [34]. This diversity is the conceptual basis in the iterative SELEX (Systematic Evolution of Ligands by EXponential enrichment) process to identify high-affinity binders. Based on our previous studies, we have shown that during a typical SELEX process, aptamers with a high GC content tend to prevail, and the melting temperature (Tm) of the aptamers gradually increases in subsequent rounds of selection [35,36,37,38,39,40,41,42,43,44]. In light of these findings, we specifically designed aptamer libraries that align with these characteristics by targeted mutagenesis. We revisited the well-established dITP-based epPCR method 33 years after its introduction by Oskar Kuipers, truly qualifying this method, without any attitude of disrespect, as an “Old Hat”. We used it for initiating SELEX, leveraging its ability to create genetic diversity from a single aptamer template, thereby showing and appreciating the usefulness of this “Old Hat” method in modern aptamer (sensing) technology.

**Figure 2:**
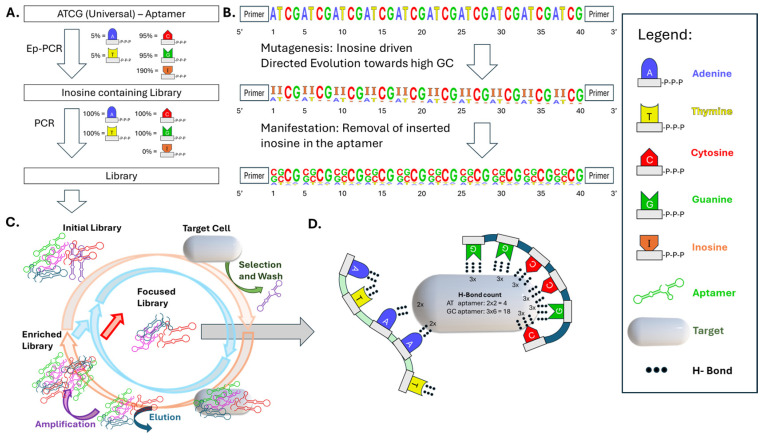
A schematic overview of inosine-mediated error-prone PCR for aptamer library generation and selection. (**A**) Overview of library creation: EpPCR introduces inosines (I) into the aptamer sequence, which are then converted into a focused mutation library during subsequent PCR cycles. (**B**) Mechanism of inosine mutagenesis: Inosines are incorporated randomly and preferentially mutated toward G/C bases, increasing the GC content and leading to sequence diversification. (**C**) Integration into SELEX: The initial library undergoes iterative cycles of selection, washing, elution, and amplification, enriching for high-affinity aptamers. (**D**) Impact of GC enrichment: An increased GC content leads to a higher number of hydrogen bonds in aptamer–target interactions, enhancing binding strength.

By applying inosine-mediated error-prone mutagenesis, we demonstrate how this approach effectively generates functional aptamer libraries capable of undergoing selection cycles. In this method, a single-stranded DNA aptamer is subjected to mutagenesis using deoxyinosine triphosphate (dITP) during PCR. Inosine acts as a universal base during amplification and is preferentially converted into guanine or cytosine in the subsequent amplification, thereby increasing GC content and introducing focused mutations [45] (Figure 2). These changes not only diversify the sequence pool but also enhance thermal stability and structural rigidity due to the formation of stronger GC base pairs, which can support more stable secondary structures and allow for an increased number of hydrogen bonds with target molecules. The resulting library is then amplified with high-fidelity polymerase and converted into a single-stranded form for use in whole-cell SELEX. In the early rounds, the affinity of the aptamers steadily increases as the sequence pool is enriched for higher-affinity binders. However, after several rounds, a point is reached where the selection efficiency begins to plateau, and further rounds yield minimal improvements in affinity. This phenomenon is observable basically in every SELEX-based study and we dare to suggest referring to this in the future as the “critical rounds” in SELEX processes in general. In this stage, most of the high-affinity aptamers have already been selected, and continued rounds of SELEX no longer lead to significant enhancement in binding strength. Understanding the timing of the critical round is essential for optimizing the SELEX process, as excessive rounds beyond this point can be inefficient, resulting in unnecessary resource expenditure without a corresponding gain in the quality of the aptamers. By this mutagenesis strategy, we accelerate the enrichment of high-affinity binders in the early SELEX rounds, where affinity increases significantly. By introducing focused mutations that enhance GC content, taking a shortcut to higher GC content ensures that aptamers with strong binding capabilities are selected earlier in the process. As a result, the library quickly converges towards the high-affinity aptamers, reaching the critical rounds faster. In summary, inosine-enhanced epPCR can represent an actual replacement of the use of commercial aptamer starting libraries and may in consequence inspire aptamer researchers to simplify the SELEX procedure, thereby opening new avenues towards further consolidation of the aptamer technology for the development of alternative powerful binding molecules.

## 2. Materials and Methods

### 2.1. Cells and Culture Conditions

The bacteria strains *R. intestinalis* (DSM-14610), *P. distasonis* (DSM-29491), *A. muciniphila* mucT (DSM-22959), *B. producta* (DSM-29491), and *R. microfusus* (DSM-15922) were cultivated in Schaedler-Bouillon Medium (Carl Roth GmbH + Co. KG, Karlsruhe, Germany) at 37 °C under anaerobic conditions.

*Escherichia coli* (strain DH5α) and *Pseudomonas aeruginosa* (strain PAO1) were cultivated in Luria–Bertani (LB) medium under aerobic conditions. Single colonies from freshly streaked LB agar plates were inoculated into 5 mL of LB broth and incubated overnight at 37 °C with constant shaking at 200 rpm. For experimental use, overnight cultures were diluted 1:100 in fresh LB medium and grown at 37 °C with shaking at 200 rpm until reaching the end of the logarithmic growth phase (OD_600_ ≈ 0.8–1.0).

The human pancreatic cancer cell line Mia PaCa-2, the lung adenocarcinoma cell line A549, and the breast cancer cell lines MDA-MB-231 and MCF-7 were cultured under standard conditions at 37 °C in a humidified atmosphere containing 5% CO_2_.

Mia PaCa-2 cells were maintained in Dulbecco’s Modified Eagle Medium (DMEM; Gibco, Waltham, MA, USA) supplemented with 10% fetal bovine serum (FBS; Sigma-Aldrich, St. Louis, MO, USA), 2.5% horse serum (Gibco), and 1% penicillin–streptomycin (Pen/Strep; Gibco).

A549 cells were cultured in DMEM supplemented with 10% FBS and 1% Pen/Strep.

MDA-MB-231 cells were grown in RPMI-1640 medium (Gibco) supplemented with 10% FBS and 1% Pen/Strep.

MCF-7 cells were maintained in Minimum Essential Medium (MEM; Gibco) supplemented with 10% FBS, 1% non-essential amino acids (NEAA; Gibco), 1 mM sodium pyruvate (Gibco), 10 μg/mL of insulin (Sigma-Aldrich), and 1% Pen/Strep.

Cells were passaged at approximately 80% confluence using Accutase (Sigma-Aldrich) according to the manufacturer’s instructions. Detached cells were centrifuged at 300× *g* for 5 min, resuspended in fresh medium, and counted using a Neubauer counting chamber. For experiments, 20,000 cells per well were seeded in 96-well flat-bottom plates (TPP) and allowed to adhere and grow for 24 h prior to further treatment. Culture media were refreshed every 2–3 days. Cells were used at passage numbers between P4 and P20.

#### Cell Pretreatment

Prior to experimental application, bacterial cultures were harvested by centrifugation at 9000× *g* for 5 min at room temperature and washed three times with sterile phosphate-buffered saline (PBS; Gibco). After the final wash, bacterial pellets were resuspended in sterile Dulbecco’s PBS (DPBS; Gibco) and adjusted to an optical density (OD_600_) of 1.0.

Eukaryotic cells were detached using Accutase (Sigma-Aldrich) according to the manufacturer’s instructions. After detachment, cells were allowed to recover in culture medium for 20 min. Cells were then washed three times with DPBS by centrifuging at 200× *g* in an Eppendorf reaction tube. The cell pellet was resuspended in fresh medium prior to further experimental use.

### 2.2. PCR Conditions

#### 2.2.1. Error-Prone PCR

For mutagenesis, 90% of the desired nucleotide was substituted with deoxyinosine triphosphate (dITP), and PCR was performed using Taq polymerase. Depending on the desired effect on melting temperature, either cytosine and guanine were replaced with inosine to reduce GC content and lower the melting temperature, or adenine and thymine were replaced to increase GC content and raise the melting temperature.

A total of 9.25 μL of EpPCR master mix was pipetted into a full-skirt 96-well PCR plate. Subsequently, 1 μL of the aptamer template at a concentration of 0.1 ng/μL was added to each well. For each mutagenesis reaction, a positive control (without dITP) and a negative no-template control were included.

Quantitative PCR (qPCR) was performed using the qTower^3^ G system, beginning with an initial denaturation step at 94 °C for 3 min. This was followed by up to 40 amplification cycles, each consisting of

Denaturation at 94 °C for 30 s;

Annealing at 56 °C for 30 s;

Elongation at 72 °C for 10 s.

SYBR Green fluorescence was measured at the end of each elongation step using excitation at 470 nm and emission at 520 nm. The fluorescence data were plotted in real time.

After completion of the 40 cycles, a melting curve analysis was performed by increasing the temperature from 60 °C to 94 °C in 1 °C increments per minute. At each step, fluorescence was measured after thermal equilibrium was reached. The first derivative of the absolute value of the melting curve was plotted using Jena Analytik qPCR software 4.0.

After qPCR, the reaction products were transferred into sterile 1.5 mL tubes and stored at −20 °C for further analysis.

#### 2.2.2. PCR Conditions

The concentration of the error-prone PCR (epPCR) product was measured using a NanoPhotometer N60 (Implen GmbH, München, Germany) and subsequently diluted to 0.1 ng/μL. These diluted samples were then amplified by qPCR using a reaction mixture containing all four nucleotides.

For amplification with Herculase II Fusion polymerase, a hot-start protocol was applied. The initial denaturation was performed at 94 °C for 3 min, followed by up to 40 cycles of

Denaturation at 94 °C for 30 s;

Annealing at 54 °C for 30 s;

Elongation at 72 °C for 10 s.

SYBR Green fluorescence was measured after each elongation step using an excitation wavelength of 470 nm and emission at 520 nm. The fluorescence intensity was plotted in real time to monitor qPCR amplification.

A melting curve analysis was performed in the same manner as after the initial epPCR: the temperature was increased from 60 °C to 94 °C in 1 °C increments per minute. At each step, SYBR Green fluorescence was measured once thermal equilibrium was reached. The first derivative of the absolute value of the melting curve was plotted using Jena Analytik qPCR software 4.0.

#### 2.2.3. PCR Cleanup

After each PCR, the products were purified using the MACHEREY-NAGEL NucleoSpin^®^ Gel and PCR Clean-up kit (MACHEREY-NAGEL GmbH & Co. KG, Düren, Germany). A master mix was prepared by combining one volume of PCR product with two volumes of NTC buffer for single-stranded DNA (ssDNA) or NTI buffer for double-stranded DNA (dsDNA). Three volumes of 2-propanol were added to this mixture. Additionally, 10 μL of sodium acetate was added for every 700 μL of master mix. The solution was mixed thoroughly.

To bind the DNA, 700 μL of the master mix was loaded onto a NucleoSpin^®^ Gel and PCR Clean-up column and centrifuged at 11,000× *g* for 1 min. The flow-through was collected and re-applied to the column twice more to ensure maximum DNA binding.

The silica membrane was then washed twice with 700 μL of NT3 buffer, centrifuging each time for 1 min at 11,000× *g*.

To dry the membrane, the column was transferred to a new collection tube and centrifuged for 1 min at 11,000× *g*. For elution, 30 μL of RNase-free water was pipetted onto the membrane. The column was incubated at room temperature for 3 min before centrifugation for 1 min at 11,000× *g*. The eluted DNA was collected in a sterile 1.5 mL tube for downstream applications.

#### 2.2.4. Strand Separation

Enzymatic strand separation was performed by incubating 44 μL of 5′-phosphorylated linear double-stranded DNA (dsDNA) aptamer—containing up to 5 μg of DNA—with 5 μL of 10× Lambda Exonuclease Reaction Buffer and 1 μL (5 units) of Lambda Exonuclease. The reaction was carried out at 37 °C for 30 min with orbital shaking at 100 rpm. Enzyme activity was terminated by heat inactivation at 75 °C for 10 min. The resulting product was purified using the PCR clean-up protocol described above.

#### 2.2.5. Primers

5′-Cy5-labeled forward primer ([Cy5]-TAGGGAAGAGAAGGACATATGAT) and phosphorylated reverse primer (Phosphate-TCAAGTGGTCATGTACTAGTCAA), both synthesized by biomers.net GmbH (Ulm, Baden-Württemberg, Germany), were used for all PCRs.

#### 2.2.6. SELEX Library Control

The used ssDNA aptamer library with 40 randomized base pairs was purchased from TriLink Biotechnologies (San Diego, CA, USA).

### 2.3. Binding Assays

To determine the affinity of the generated aptamers, binding assays were performed. An amount of 30 pmol of Cy5-labeled aptamers was first activated by heating at 90 °C for 5 min, followed by rapid cooling to 4 °C and subsequent equilibration to 25 °C.

The activated aptamers were then incubated with 10^8^ procaryotic or 20,000 eukaryotic cells for 30 min on a rotator at 50 rpm, at room temperature in the dark. Following incubation, three washing steps were performed using 500 μL of DPBS per step. The cells were centrifuged at 8000× *g* or 200× *g* (eukaryotic cells) for 2 min after each wash to remove unbound aptamers.

After the final wash, the cells were resuspended in 200 μL of DPBS and lysed by heating at 95 °C for 5 min to release the bound aptamers. The lysates were centrifuged at 10,000× *g* for 2 min, and the supernatants were collected for fluorescence analysis.

For fluorescence measurement, the supernatants containing the released aptamers were transferred to a Luminox 384-well fluorescence plate. Fluorescence intensity was measured using a Tecan Infinite M200 (Tecan Group Ltd., Männedorf, Switzerland) and a Tecan Spark (Tecan Group Ltd., Männedorf, Switzerland) plate reader at an excitation wavelength of 635 nm and an emission wavelength of 670 nm.

### 2.4. Next-Generation Sequencing

The three initial libraries were analyzed by Illumina^®^ sequencing by Eurofins genomics (Konstanz, Germany). Since Illumina sequencing requires bridge amplification, the aptamer sequences were extended via two rounds of PCR to incorporate the necessary adapter regions.

In the first PCR step, universal primer binding sites were introduced. The resulting extended double-stranded aptamers were purified using the MACHEREY-NAGEL NucleoSpin^®^ Gel and PCR Clean-up kit.

In the second PCR, index sequences were incorporated to enable multiplexed sequencing.

The resulting sequencing data was processed using the usegalaxy suite (https://usegalaxy.org/). Downstream sequence analysis was conducted using the FASTAptamer toolkit (Bond Life Sciences Center, Columbia, MO, USA, https://burkelab.missouri.edu/fastaptamer.html) See Supplementary Data for raw sequencing files.

## 3. Results

**Figure 3:**
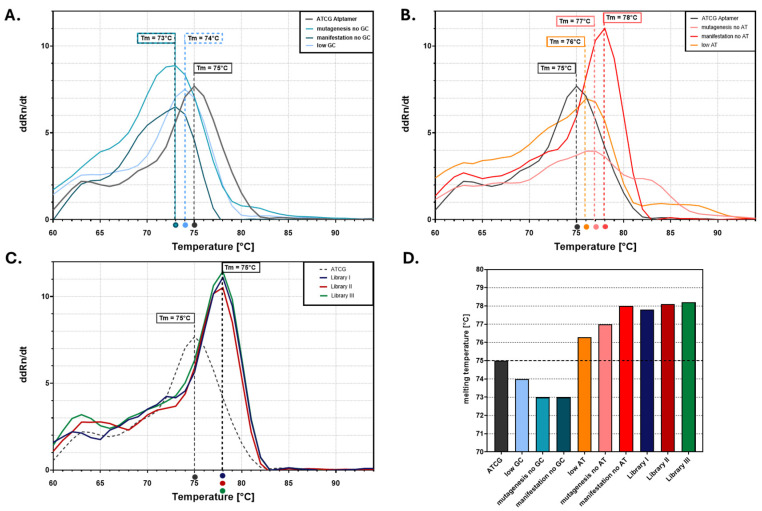
(**A**–**C**) Derivative melting curves (d(dR)/dT vs. Temperature) of the ATCG aptamer and its sequence variants or libraries were measured to assess thermal stability. (**A**) Variants with mutations in GC base pairs and generally reduced GC content exhibit lower melting temperatures (Tm), indicating decreased thermal stability. The curve labeled “Mutagenesis no GC” corresponds to an inosine-containing aptamer library in which approximately 90% of GC pairs were replaced by I during the PCR step. The “Manifestation no GC” curve represents the same library after a follow-up PCR using standard nucleotides (A, T, G, and C), thereby removing inosine and reflecting the manifest sequence diversity induced by the original inosine substitutions. (**B**) Variants with mutations on AT pairs and overall low AT content exhibit higher Tm values, suggesting increased stability. The curve labeled “Mutagenesis no AT” corresponds to an inosine-containing aptamer library in which approximately 90% of GC pairs were replaced by I pairs during the PCR step. The “Manifestation no AT” curve represents the same library after a follow-up PCR using standard nucleotides (A, T, G, and C), thereby removing inosine and reflecting the manifest sequence diversity induced by the original inosine substitutions. (**C**) Aptamer libraries (Libraries I–III) were generated by mutagenesis and subsequent manifestation with the substitution of 90% GC for standard nucleotides. They display distinct melting behavior compared to the wild-type ATCG, with all showing increased Tm values. Libraries I–III were generated using the same mutagenesis protocol and represent independent replicates. (**D**) A bar graph summarizing the melting temperatures (from (**A**–**C**)) for all aptamer variants and libraries, showing a trend of increased thermal stability with reduced AT content and in pooled libraries. Statistical analysis was thus not applicable.

Inosine-mediated epPCR mutagenesis was performed by partially replacing canonical nucleotides with deoxyinosine triphosphate (dITP) during PCR (mutagenesis), intended to result in the random incorporation of inosines. A follow-up PCR with A, T, C, and G represented equally (manifestation) led to the preferential conversion of previously incorporated inosines into either guanine and cytosine or adenine and thymine bases during subsequent amplification cycles. For comparison, an additional mutagenesis reaction served as a control using a low-fidelity Taq polymerase with a nucleotide imbalance without dITP, yielding a control library labeled low AT/low GC. Melting curve analysis provided a first indication of successful mutagenesis and sequence divergence (Figure 3). Aptamer variants generated by reducing the GC content (Figure 3A) showed lower melting temperatures (Tm), while those with increased GC content (Figure 3B) exhibited higher Tm values, reflecting greater thermal stability. In contrast, the controls labeled low AT or low GC mutagenesis alone exhibited a less pronounced shift and lower overall sequence divergence relative to the inosine-based approach; thus, the libraries with the higher error rates were selected. Notably, all three epPCR-derived libraries (Libraries I–III) (Figure 3C) showed distinct and elevated melting profiles compared to the wild-type ATCG aptamer, suggesting not only a shift in base composition but also a consistency of the applied method (Figure 3D). All three libraries (Libraries I–III) were generated independently using the same mutagenesis protocol and thus represent independent replicates of this experiment.

**Figure 4:**
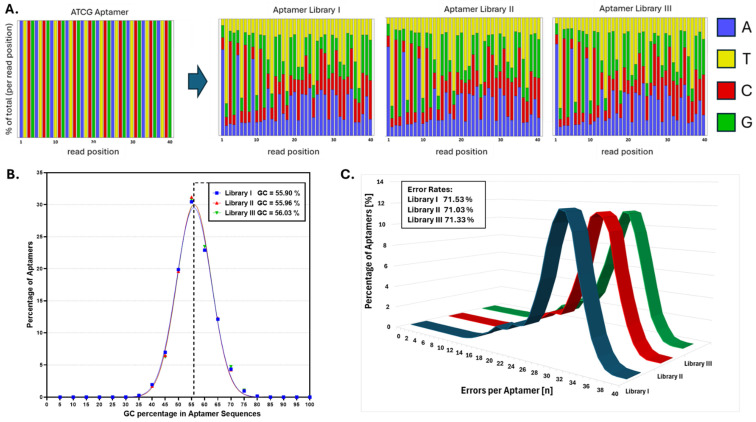
The sequence diversity and mutation analysis of ATCG aptamer libraries. (**A**) The nucleotide distribution across read positions for the original ATCG aptamer and the three variant libraries. The ATCG aptamer shows a uniform and fixed sequence, while Libraries I–III display increased nucleotide diversity at each position, indicating successful mutagenesis. (**B**) The distribution of GC content across aptamer sequences in each library. Libraries I–III show a narrow, symmetric distribution centered around ~55–56% GC content. (**C**) A histogram of total errors (mutations) per aptamer for each library. Libraries I–III show a similar range and distribution of sequence errors, with overall mutation rates of ~71%.

Next-generation sequencing (Figure 4) confirmed high mutation efficiency and sequence diversity across all libraries. The nucleotide distribution plots showed that nearly every position in the original ATCG aptamer had undergone diversification, yielding pools with broad sequence heterogeneity (Figure 4A). The GC content distributions for each library were narrow and symmetrically centered around ~55–56%, consistent with the preferential transition of inosines to guanines and cytosines during subsequent PCR amplification (Figure 4B). The average mutation load per aptamer was similar across all libraries, around 71%, suggesting a certain reproducibility of the mutagenesis protocol. Furthermore, the singleton ratios, defined as the proportion of sequences appearing only once in the dataset, were 33.31% for Library I, 33.47% for Library II, and 31.49% for Library III.

**Figure 5:**
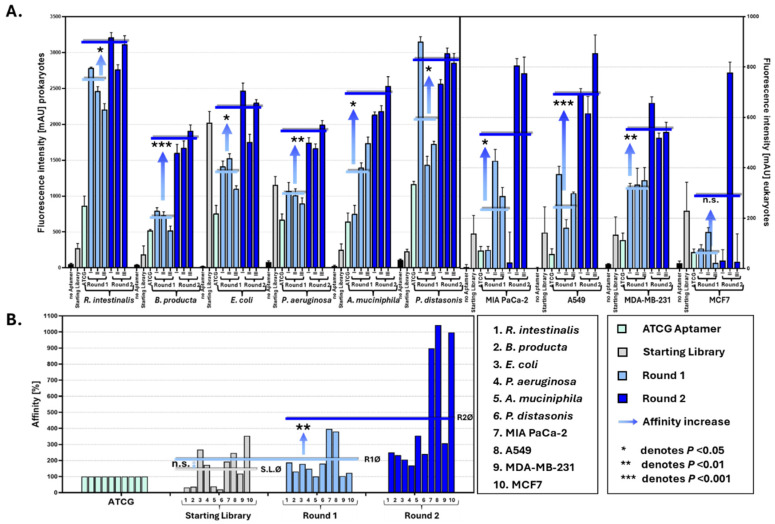
The functional screening and affinity improvement of aptamer libraries against diverse bacterial and cancer cell targets. (**A**) The fluorescence intensity of aptamer binding to ten different target cell types (bacterial and cancer cells), comparing the original ATCG aptamer, a randomly synthesized starting aptamer library with balanced nucleotide representation, and Round 1-enriched and Round 2-enriched libraries. Data show significantly increased fluorescence intensities after each round of selection, indicating enhanced binding affinities (* *p* < 0.05, ** *p* < 0.01, *** *p* < 0.001). (**B**) The normalized aptamer affinity relative to the original ATCG aptamer across targets.

To evaluate whether this sequence diversity really translated, as intended, into a “gain of function”-generating binding competent aptamers with considerable specificity towards the dedicated targets, we conducted binding assays after one and two selection rounds against a panel of ten targets including five bacterial strains and five cancer cell lines (Figure 5). Fluorescence-based affinity measurements showed significant increases in target binding after each selection round. On average, aptamer affinity increased by 162.2% after the first round and by 491.7% after the second compared to the wild-type ATCG aptamer, demonstrating that the libraries were not only diverse but in fact also capable of a rapidly evolving enhanced target affinity under selection pressure (Figure 5B).

## 4. Discussion

The SELEX process as the key methodology in current aptamer research typically relies on the use of large (commercially available) libraries of synthetic oligonucleotides with randomized sequences of different lengths (depending on the design of the library used) [46,47,48]. As a so-called FluCell-SELEX, it is extremely feasible to isolate polyclonal or focused aptamer libraries against eukaryotic and procaryotic cells (e.g., pathogenic yeasts, probiotic or pathogenic bacteria) which then in turn can serve as the “evolved” productive sequence space from which high-performing individual aptamers can be selected [49,50,51] for a variety of applications in biotechnology and medicine including the development of electronic aptasensor chips [52,53,54]. The rationale behind the study presented here was to evaluate whether a basic epPCR method with proven power in creating diversity from given starting sequences could develop enough diversity from an oligo-ATCG-repeat DNA-oligonucleotide in a very limited number of reactions to serve as the molecular basis for diversity evolution (“molecular breeding”). A desired additional technological aspect was to eventually reduce economic burdens in SELEX-dependent studies of purchasing costly randomized initial libraries. The results presented in this study not only show that, based on a single round of inosin-mediated-epPCR-mutagenesis and two subsequent selection rounds resembling the typical critical rounds of a SELEX process, it delivers a considerable gain of affinity against all cellular targets, but also demonstrate the general applicability of this well-established epPCR method. We hope that the ease of this “Old Hat” directed evolution method may inspire aptamer scientists to not only optionally replace commercial initial libraries as the starting resource for SELEX processes but to also include this quite simple method in the SELEX rounds to increase selection pressure. This may open new avenues towards more efficient SELEX strategies not only in terms of experimental speed but also of economic affordability.

## 5. Conclusions

Inosine-mediated error-prone PCR provides a simple, cost-effective strategy for generating diverse and functional aptamer libraries from a single sequence. Our results show that these libraries can undergo successful selection within just two SELEX rounds, yielding aptamers with significantly improved binding affinities across diverse cell targets. This study highlights how revisiting established techniques can empower modern molecular tools and lower the barrier to aptamer discovery.

## Data Availability

The data can be requested from the authors.

## Supplementary Materials

The following supporting information can be downloaded at https://cloudstore.uni-ulm.de/s/cW8QXfdHZgpn5Ff (accessed on 10 March 2025).
